# Calreticulin mediates immune recognition of acute myeloid leukemia cells in vivo

**DOI:** 10.1186/2051-1426-1-S1-P148

**Published:** 2013-11-07

**Authors:** Xiufen Chen, Dominick Fosco, Douglas E Kline, Justin Kline

**Affiliations:** 1 Department of Medicine, University of Chicago, Chicago, IL, USA; 2 University of Chicago Comprehensive Cancer Center and Committee on Immunology, University of Chicago, Chicago, IL, USA

## 

Calreticulin (CRT) is a chaperone protein which normally resides in the endoplasmic reticulum (ER). However, recent studies have demonstrated that pre-apoptotic cancer cells release internalized CRT to their surface prior to death, and this surface exposure of CRT acts as an ‘eat-me’ signal to local phagocytes. Some chemotherapeutic agents and gamma-irradiation have been shown to induce ER stress and promote CRT surface translocation in cancer cells, resulting in their recognition and phagocytosis by innate immune cells, such as macrophages and dendritic cells (DC). In turn, innate immune cells which have phagocytized CRT-expressing tumor cells become capable of priming antigen-specific T cell responses directed against malignant cells. Unfortunately, chemotherapy and irradiation which can induce immunogenic cell death (ICD) through CRT, can also result in local and/or systemic immune suppression in the host. To bypass the requirement of exposing the host to chemotherapy to induce translocation of CRT to the cell surface, C1498 AML cells were engineered to constitutively express cell surface CRT (C1498.CRT). Introduction of C1498.CRT cells subcutaneously (SC) into syngeneic C57BL/6 mice resulted in either significantly delayed tumor outgrowth or complete protection from tumor development compared to parental C1498 cells which universally progressed in vivo. A SC challenge with C1498.CRT cells also protected mice from tumor outgrowth following a subsequent re-challenge with parental C1498 tumor cells, suggesting that C1498.CRT cells promote immunologic memory. Differences in tumor outgrowth between mice inoculated with parental C1498 and C1498.CRT leukemia cells were abrogated in immunodeficient RAG-/- and RAG2-/-γc-/- animals, arguing that the immune-mediated effect of cell-surface CRT expression is dependent upon a functional adaptive immune system. To model a clinically relevant scenario, parental C1498 or C1498.CRT cells were inoculated intravenously (IV) into C57BL/6 mice. Significantly prolonged survival was observed in hosts harboring C1498.CRT versus parental C1498 cells systemically. Systemic inoculation with C1498.CRT cells expressing the model SIYRYYGL (SIY) peptide antigen (C1498.SIY.CRT cells) resulted in enhanced expansion and effector cytokine production by antigen-specific T cells compared to T cells from hosts challenged with control C1498.SIY cells. Our current on-going experiments are focusing on identifying the mechanism(s) through which calreticulin appears to promote anti-tumor immunity.

